# 1,2-Dichloropropane, but not dichloromethane or trichloropropane, reduces apoptosis of human cholangiocytes co-cultured with macrophages

**DOI:** 10.1093/joccuh/uiag029

**Published:** 2026-06-01

**Authors:** Mst Mahfuza Rahman, Kyoshiro Kusagaya, Cai Zong, Yusuke Kimura, Abigail Ekuban, Ryoya Takizawa, Daichi Nagashima, Alzahraa Fergany, Sahoko Ichihara, Gaku Ichihara

**Affiliations:** Department of Occupational and Environmental Health, Faculty of Pharmaceutical Sciences, Tokyo University of Science, Katsushika, Tokyo 125-8585, Japan; Department of Pharmacy, Comilla University, Comilla 3506, Bangladesh; Department of Occupational and Environmental Health, Faculty of Pharmaceutical Sciences, Tokyo University of Science, Katsushika, Tokyo 125-8585, Japan; Department of Occupational and Environmental Health, Faculty of Pharmaceutical Sciences, Tokyo University of Science, Katsushika, Tokyo 125-8585, Japan; Department of Environmental and Preventive Medicine, Jichi Medical University School of Medicine, Shimotsuke 329-0498, Japan; Department of Occupational and Environmental Health, Faculty of Pharmaceutical Sciences, Tokyo University of Science, Katsushika, Tokyo 125-8585, Japan; Department of Environmental and Preventive Medicine, Jichi Medical University School of Medicine, Shimotsuke 329-0498, Japan; Yokohama University of Pharmacy, Japan; Department of Occupational and Environmental Health, Faculty of Pharmaceutical Sciences, Tokyo University of Science, Katsushika, Tokyo 125-8585, Japan; Laboratory of Genetics and Genetic Engineering in Department of Animal Husbandry and Animal Wealth Development, Faculty of Veterinary Medicine, Alexandria University, Alexandria, Egypt; Department of Environmental and Preventive Medicine, Jichi Medical University School of Medicine, Shimotsuke 329-0498, Japan; Department of Occupational and Environmental Health, Faculty of Pharmaceutical Sciences, Tokyo University of Science, Katsushika, Tokyo 125-8585, Japan

**Keywords:** 1,2-dichloropropane, dichloromethane, 1,2,3-trichloropropane, cholangiocarcinoma, occupational cancer, DNA damage, carcinogenicity, anti-apoptotic effect

## Abstract

**Objectives:**

The roles of dichloromethane (DCM) and 1,2,3-trichloropropane (1,2,3-TCP) in occupational cholangiocarcinoma, first reported in 2012, remain elusive. This study aimed to determine the potential of 1,2-dichloropropane (1,2-DCP), DCM, and 1,2,3-TCP to induce DNA damage—a hallmark of carcinogenicity—in human cholangiocytes.

**Methods:**

Monocultures of human immortalized cholangiocytes (MMNK-1 cholangiocytes) and co-cultures of MMNK-1 cholangiocytes with human THP-1 monocyte-derived macrophages and monocytes were each exposed to 1,2-DCP, DCM or 1,2,3-TCP at 0, 0.1, and 0.4 mM for 24 hours. DNA double-strand break marker γ-H2AX-positive foci and γ-H2AX pan-nuclear staining—which is known to be induced during early or intermediate stages of apoptosis or under replicative stress/checkpoint abrogation in cholangiocytes—were counted in 100 and 200 cholangiocytes, respectively. Apoptosis was evaluated by terminal deoxynucleotidyl transferase-mediated dUTP nick end-labeling (TUNEL) staining.

**Results:**

The increase in dense (≥55) γ-H2AX foci induced by all 3 chemical compounds was significantly greater in co-cultures with macrophages, but not with monocytes, than in monocultures. However, only 1,2-DCP decreased the number of γ-H2AX pan-nuclear-positive and TUNEL-positive cholangiocytes in co-cultures with macrophages, but not in monocultured cholangiocytes. Co-treatment with a pan-caspase inhibitor decreased the number of γ-H2AX pan-nuclear-positive cholangiocytes in each group by about 50%, suggesting that apoptotic signaling is involved, at least in part, in the induction of γ-H2AX pan-nuclear-positive cholangiocytes.

**Conclusions:**

Our study showed that co-culture with macrophages enhanced DNA double-strand breaks induced by all 3 tested chemical compounds, while only 1,2-DCP exhibited a proliferative effect in monocultures and anti-apoptotic effects in co-cultures with macrophages, which are key characteristics of carcinogens, thus distinguishing 1,2-DCP from DCM and 1,2,3-TCP.

## Teaser

**Key points**
 

- **What is already known on this topic:** The presence of macrophages enhances DNA damage in human cholangiocytes exposed to 1,2-dichloropropane (1,2-DCP).
- **What this study adds:** The presence of macrophages also enhances DNA damage in human cholangiocytes exposed to dichloromethane (DCM) and 1,2,3-trichloropropane (1,2,3-TCP), with 1,2,3-TCP inducing more severe DNA damage than 1,2-DCP or DCM. However, only 1,2-DCP suppresses apoptosis in cholangiocytes co-cultured with macrophages.
- **How this study might affect research, practice or policy:** This study demonstrates the anti-apoptotic effect of 1,2-DCP as a potential mechanism underlying 1,2-DCP-induced cholangiocarcinogenesis, distinguishing it from DCM and 1,2,3-TCP and providing mechanistic evidence for its carcinogenicity.

## 1. Introduction

By June 2013, 17 workers at an offset proof printing company in Osaka, Japan, had been diagnosed with either intra- or extra-hepatic cholangiocarcinoma.^1^^,^^2^ Subsequent investigations of other Japanese printing companies also identified cases of bile duct cancer related to chemical exposure.^3^^,^^4^ Based on these reports, it has been assumed that these occupation-related cancers are linked to workplace chemical factors.^1^^,^^2^ The workers diagnosed in the above studies developed cholangiocarcinoma at a younger age (25-45 years) compared with the average age of patients in the regional cholangiocarcinoma registry.^5^ Histopathological examination of surgical specimens from the affected workers showed pre-cancerous and early cancerous lesions, including biliary intraepithelial neoplasia and intraductal papillary neoplasia in the bile duct, in addition to bile duct sclerosis, inflammatory cell infiltration, bile duct epithelial injury/focal bile duct defects, and bile duct epithelial hyperplasia at various locations in the noncancerous liver tissue.^5^

A follow-up survey of the working environment showed the use of 1,2-dichloropropane (1,2-DCP) from 1985 to 2006 and dichloromethane (DCM) from 1985 to 1996 in these factories, mainly in the process of ink removal from the transfer rubber rollers.^6^ Since 1,2-DCP was the sole chemical to which all of the workers who developed cholangiocarcinoma were exposed, it was considered to be the causative agent, although those investigations did not rule out completely the possible involvement of DCM.^3,6^ Based on early studies of the carcinogenicity and genotoxicity of dihalogenated hydrocarbons, such as 1,2-dichloroethane or 1,2-dibromoethane, which are structurally similar to 1,2-DCP, it was hypothesized that the carcinogenic effects of 1,2-DCP might be mediated through the formation of the episulfonium ion and subsequent induction of DNA adducts. However, previous murine studies had shown that 1,2-DCP did not induce the formation of episulfonium ions.^7^

Carcinogenicity studies in rats, mice, and hamsters have also shown the potential susceptibility to DCM-induced malignancies in mice only (lung and liver) following exposure to 1000-4000 ppm.^8^ Following the suspicion of 1,2-DCP and DCM in the causation of occupational cholangiocarcinoma, the International Agency for Research on Cancer (IARC) reclassified 1,2-DCP from Group 3 (not classifiable as carcinogenic to humans) to Group 1 (carcinogenic to humans) as well as DCM from Group 2B (possibly carcinogenic to humans) to Group 2A (probably carcinogenic to humans) in 2014.^9^

Apart from the printing factories, the above chemicals have also been reported to be involved in other malignancies and serious pathologies. Prior to the 1980s, soil fumigants containing chloropropane were extensively used in US agriculture as pesticides and nematocides. Soil fumigants composed primarily of 1,3-dichloropropene and 1,2-DCP, with minor concentrations of 1,2,3-trichloropropane (1,2,3-TCP), were commercially promoted for agricultural use across a range of crops.^10^ Toxicological studies in mice exposed to 1,2,3-TCP for 2 years showed the development of forestomach tumors with a mutation signature almost identical to that of cholangiocarcinoma found in the offset printing workers.^11^ Other studies showed that exposure of male and female guppies to 1,2,3-TCP induced liver tumors.^12^ In aqueous studies, exposure to 1,2,3-TCP increased the incidence of various liver tumors and gallbladder papillary adenomas in male and female medaka (Japanese rice fish).^13^ Lastly, a recent case report described the development of severe liver damage in a Chinese individual studying at the chemical engineering faculty following exposure to 1,2,3-TCP.^14^

The present study was designed to determine the harmful effects of 3 chlorinated hydrocarbons (1,2-DCP, DCM and 1,2,3-TCP) on DNA structure in cultured human cholangiocytes.

## 2. Materials and methods

### 2.1 Cell lines and cell cultures

Human immortalized cholangiocytes (MMNK-1 cholangiocytes) were cultured in Dulbecco’s modified Eagle’s medium, supplemented with low glucose (DMEM, FUJIFILM Wako Pure Chemical Industries, Osaka, Japan) and 5% heat-inactivated fetal bovine serum (FBS, Lot #S17692S1820; BioWest, Riverside, MO, USA), at 37°C under 5% CO_2_. Human monocyte lineage cells (THP-1 cells) were cultured in Roswell Park Memorial Institute medium 1640 (RPMI 1640; Wako, Japan) supplemented with 10% heat-inactivated FBS, penicillin, streptomycin, l-glutamine (Gibco, Thermo Fisher, Waltham, MA, USA) and 2-mercaptoethanol (0.05 mM; Sigma Aldrich, St Louis, MO, USA) at 37°C under 5% CO_2_. Based on the importance of hepatic macrophages (including resident Kupffer cells and infiltrating monocyte-derived macrophages) in liver inflammation and fibrosis,^15^ we also added macrophages as inflammatory cells in the MMNK-1/THP-1 co-culture model. The cell seeding density was selected based on a previous study conducted in our laboratory.^16^ Specifically, THP-1 cells were differentiated on cell culture inserts (Corning Inc, Corning, NY, USA) with a 0.4 μm pore size membrane for 48 hours using 162 nM phorbol 12-myristate 13-acetate (PMA, Sigma-Aldrich). Next, the differentiated THP-1 macrophages on the inserts were washed with phosphate-buffered saline (PBS). We also prepared undifferentiated THP-1 monocytes on inserts to determine the effect of differentiation from monocytes into macrophages. The inserts were combined with culture plates on which MMNK-1 cholangiocytes were pre-cultured for 12 hours after seeding. Co-cultures of MMNK-1 cholangiocytes and differentiated THP-1 macrophages or undifferentiated THP-1 monocytes were maintained in a 1:1 ratio of DMEM and RPMI 1640 mixed medium with 5% FBS. Following their incubation for 12 hours, the co-cultured cells were exposed to different concentrations of 1,2-DCP, DCM, and 1,2,3-TCP for 24 hours.

### 2.2 Cell exposure to 1,2-DCP, DCM, and 1,2,3-TCP

1,2-DCP (98% purity), DCM (99.5% purity), and 1,2,3-TCP (99% purity) were purchased from FUJIFILM Wako Pure Chemical Corporation. They were first mixed with dimethyl sulfoxide (DMSO; FUJIFILM Wako Pure Chemical) to enhance their dispersibility and then diluted in a culture medium at the desired concentrations for use in cell cultures. The concentration of DMSO was adjusted to 0.1% for both the control and exposure groups. During exposure to 1 of the 3 chlorinated hydrocarbons, the seeded cells were sealed in Tedlar polyvinyl fluoride (PVF) gas sampling bags, as described previously.^17^ The concentrations used in the present study were based on data available in previous studies.

The estimated exposure concentrations of 1,2-DCP and DCM at the sites of industrial cholangiocarcinoma ranged from 100 to 670 and 80 to 540 ppm, respectively.^1^^,^^2^ The blood 1,2-DCP and DCM concentrations in equilibrium with 1,2-DCP and DCM vapor in alveoli (ppm [v/v]) were estimated with a blood:air partition of 10.7.^1^^,^^2^^,^^11^ Alveolar 1,2-DCP and DCM at 1000 ppm are in equilibrium with approximately 0.4 mM 1,2-DCP in blood (1,2-DCP: 1 ppm = 4.62 mg/m^3^; DCM: 1 ppm = 3.53 mg/m^3^).^6^ Based on the above data, the selected concentrations of 1,2-DCP and DCM used in the present study ranged from 0 to 0.8 mM. The selected exposure concentration of 1,2,3-TCP ranged from 0 to 0.8 mM to allow comparison of the cytotoxicity of 1,2-DCP and DCM.

### 2.3 MTS assay

The MTS cell viability assay was performed on monocultured MMNK-1 cholangiocytes (96-well plate: 1.0 × 10^4^ cells/well) and MMNK-1 cholangiocytes (24-well plate: 3.0 × 10^4^ cells/well) co-cultured with THP-1-derived macrophages (culture inserts for 24-well plate: 6.0 × 10^4^ cells/well) (hereafter termed cholangiocyte/macrophage co-cultures). After a 12-hour preincubation of cholangiocytes/macrophages as described in the co-culture method section and 24 hours for monocultured MMNK-1 cells (hereafter termed mono-cholangiocytes), the cells were exposed to 0, 0.05, 0.1, 0.2, 0.4, and 0.8 mM 1,2-DCP, DCM, or 1,2,3-TCP for 24 hours. Cell viability was quantified post-exposure using the CellTiter 96® AQueous One Solution Cell Proliferation Assay (Promega, Madison, WI, USA). Absorbance at 490 nm was measured with a PowerWave XS2 microplate reader (BioTek, Winooski, VT, USA).

### 2.4 Immunostaining for localization of **γ**-H2AX-positive MMNK-1 cholangiocytes

Immunocytochemistry was employed for localization of γ-H2AX, a DNA double-strand break marker,^18^^,^^19^ in the nuclei of cholangiocytes. Immunostaining of γ-H2AX-positive foci was performed on mono-cholangiocytes (2.5 × 10^4^ cells/well in 24-well plate) and cholangiocytes (2.5 × 10^4^ cells/well in 24-well plate) co-cultured with macrophages or undifferentiated monocytes (5.0 × 10^4^ cells/well of culture insert-loaded 24-well plate). For clear observation of the γ-H2AX foci, cholangiocytes were cultured on a sterilized 15-mm cover glass placed on the 24-well plate. After 12 hours of pre-incubation, cholangiocyte/macrophage co-cultures, cholangiocyte/monocyte co-cultures and mono-cholangiocytes were exposed to 1,2-DCP, DCM, or 1,2,3-TCP at 0, 0.1, and 0.4 mM for 24 hours.

This was followed by washing with PBS and fixation with 4% paraformaldehyde for 10 minutes at room temperature. To enhance permeability of the antibodies, 0.2% Triton X-100 was applied to the cells at room temperature for 10 minutes. Protein blocking was performed using the PBS-T buffer containing 3% BSA and 22.52 mg/mL glycine for 30 minutes at room temperature. The cells were incubated overnight with anti-phospho-histone H2A.X (Ser139) mouse Ab (#sc-517 348, dilution: 1:1000; Merck Millipore, Burlington, MA, USA) at 4°C. The cells were washed with PBS-T followed by incubation with donkey anti-mouse IgG H&L Alexa Fluor® 647 (#ab150111; Abcam, Cambridge, UK) for 1 hour at room temperature in the dark and subsequent washing with PBS-T. After the immunostaining procedure, the cover glass was removed from the 24-well plate and sealed on a slide glass with mounting medium containing 4′,6-diamidino-2-phenylindole (DAPI) (#ab104139; Abcam) for nuclear counterstaining. Then, 10-20 images per sample were selected at random using a camera attached to the fluorescence microscope.

γ-H2AX immunostaining was used to evaluate nuclear DNA double-strand break signaling. Foci were counted in 100 randomly selected cells, and cells were categorized into 3 groups based on the number of γ-H2AX foci per nucleus: γ-H2AX_sparse_ (0 to <5 foci), γ-H2AX_moderate_ (≥5 to <55 foci), and γ-H2AX_dense_ (≥55 foci) to separate groups of cells having similar kinds of distribution of foci. The marginal values of 5 and 55 were selected as they were the right tail of distribution with the smallest mode and the left tail of distribution with the largest mode, respectively, differentiating distributions with the smallest mode and the largest mode from other distributions between them (Figure S1). Because the distribution of foci was neither normal nor continuous, parametric statistical methods were not applicable. Categorization of the data enabled the use of parametric analyses, including multiple regression analysis, with commercially available software. To assess pan-nuclear γ-H2AX staining, 200 cells were selected at random and evaluated for uniform nuclear signal distribution.

### 2.5 Evaluation of role of apoptotic signaling in pan-nuclear **γ**-H2AX staining

To determine whether apoptotic signaling contributes to the appearance of pan-nuclear γ-H2AX staining (which can arise not only during early or intermediate stages of apoptosis but also in response to replicative stress or checkpoint failure^20^), we treated the cells with a pan-caspase inhibitor. Mono-cholangiocytes and cholangiocytes/macrophages were pretreated with 20 μL of pan-caspase inhibitor (VZFM-NK, Promega) for 1 hour, and then exposed to 1,2-DCP, DCM, or 1,2,3-TCP for 24 hours. Immunostaining for γ-H2AX was performed in a manner similar to that described above. The number of pan-nuclear γ-H2AX signals was counted to evaluate the effect of caspase inhibition of 1,2-DCP-, DCM-, and 1,2,3-TCP-induced γ-H2AX expression.

### 2.6 Localization of **γ**-H2AX and TUNEL staining

Monocultured cholangiocytes (24-well plate: 2.5 × 10^4^ cells/well) and cholangiocytes (24-well plate: 2.5 × 10^4^ cells/well) co-cultured with macrophages (culture inserts for 24-well plate: 5.0 × 10^4^ cells/well) were examined for cells exhibiting both γ-H2AX and terminal deoxynucleotidyl transferase-mediated dUTP nick end-labeling (TUNEL) staining to determine the extent of apoptosis. This was accomplished by counting the number of pan-nuclear γ-H2AX/TUNEL double-positive cells. After exposure to 1,2-DCP, DCM, and 1,2,3-TCP, the cells were washed with PBS and fixed with 4% paraformaldehyde for 10 minutes at room temperature. To enhance permeability, 0.2% Triton X-100 was applied to the cells for 10 minutes at room temperature. Protein was blocked with application of PBS-T buffer containing 3% BSA and 22.52 mg/mL glycine for 30 minutes at room temperature. This was followed by incubation of the cells overnight with anti-phospho-histone H2A.X (Ser139) mouse Ab (dilution: 1:1000; Merck Millipore, Burlington, MA, USA) at 4°C, followed by TUNEL staining using a TUNEL Apoptosis Detection Kit (DeadEnd Fluorometric TUNEL System; Promega, Madison, WI, USA) for in vitro detection of apoptosis, employing the instructions provided by the manufacturer. Subsequently, the TUNEL-treated cells were incubated with the secondary antibody (donkey anti-mouse IgG H&L Alexa Fluor® 647, ab150111; Abcam, Cambridge, UK) for 1 hour in the dark. For a clear examination, the cells were cultured on a sterilized 15-mm cover glass placed on the 24-well plate. After the entire staining procedure, the cover glass was taken from the 24-well plate and sealed on a slide glass with mounting medium containing DAPI. Then, 10-20 images per sample were captured by a fluorescence microscope to calculate the number of cholangiocytes with pan nuclear γ-H2AX/TUNEL double-staining. The percentage of cholangiocytes exhibiting pan-nuclear γ-H2AX/TUNEL double-positivity was calculated as follows: (number of pan-nuclear γ-H2AX/TUNEL double-positive cholangiocytes ÷ total number of TUNEL-positive cholangiocytes) × 100.

### 2.7 Statistical analysis

Data are expressed as mean ± SD. One-way analysis of variance (ANOVA) followed by Dunnett’s multiple comparison test was used to determine the effects of 1,2-DCP, DCM, and 1,2,3-TCP concentrations on cell viability (MTS assay) and γ-H2AX immunostaining γ-H2AX_sparse_ (0 to <5 foci), γ-H2AX_moderate_ (≥5 to <55 foci), and γ-H2AX_dense_ (≥55 foci). Simple regression analysis and multiple regression analysis with dummy variables were performed to evaluate the effects of chemical dose and culture type (co-culture with monocytes or macrophages to monoculture)/pan-caspase inhibitor. GraphPad Prism software (version 8.4.2 for Windows, GraphPad Software, San Diego, CA, USA) and JMP (version 19.0.3, SAS Statistical Discovery LLC, Cary, NC, USA) were used in ANOVA and simple/multiple regression analysis, respectively. A *P* value <.05 denoted statistical significance.

## 3. Results

### 3.1 DCP increases whereas TCP decreases cell viability of mono-cholangiocytes

**Figure 1:**
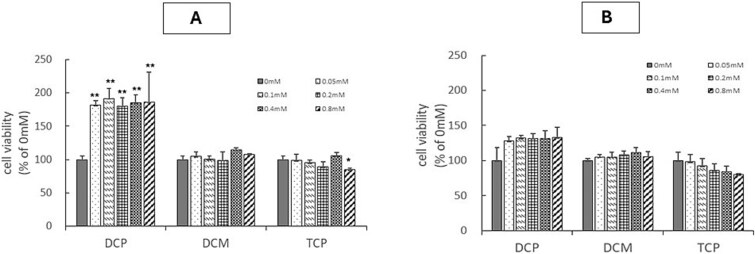
Effects of various concentrations of 1,2-dichloropropane (1,2-DCP), dichloromethane (DCM), and 1,2,3-trichloropropane (1,2,3-TCP) on cell viability of mono-cholangiocytes and cholangiocytes co-cultured with macrophages. (A) Monocultures of MMNK-1 cholangiocytes and (B) MMNK-1 cholangiocytes co-cultured with THP-1 macrophages were exposed to 1,2-DCP, DCM, and 1,2,3-TCP at final concentrations of 0, 0.05, 0.1, 0.2, 0.4, or 0.8 mM, and their cell viability was examined 24 hours later. Data are mean ± SD, *n* = 3 (*n* = number of samples). ^*^P < .05, ^**^P < .01, compared with the corresponding control group (0 mM), by 1-way analysis of variance (ANOVA) followed by Dunnett’s multiple comparison test.

Cell proliferation was quantified because it is a key driver in maintaining mutagenic DNA lesions and initiating the early phases of chemically induced carcinogenesis.^19–22^ 1,2-DCP significantly increased cell viability of mono-cholangiocytes at each concentration used in this study, compared with the control group (Figure 1A). On the other hand, DCM had no effect on cell viability of mono-cholangiocytes, compared with the control group (Figure 1A). Furthermore, at 0.8 mM, 1,2,3-TCP significantly decreased cell viability of mono-cholangiocytes, compared with the control group (Figure 1A). In contrast, the cell viability of cholangiocytes/macrophages was not affected by all 3 chlorinated hydrocarbons (Figure 1B).

### 3.2 DCP, DCM, and TCP dose-dependently increase number of **γ**-H2AX_dense_ in cholangiocytes cultured with macrophages

**Figure 2:**
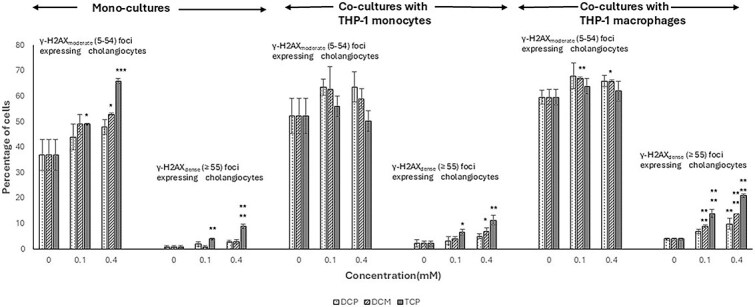
Effects of various concentrations of 1,2-dichloropropane (1,2-DCP), dichloromethane (DCM), and 1,2,3-trichloropropane (1,2,3-TCP) on γ-H2AX_moderate_ foci (5-54) and γ-H2AX_dense_ foci (≥55) in mono-cholangiocytes, cholangiocytes co-cultured with monocytes, and cholangiocytes co-cultured with macrophages. Monocultures of MMNK-1 cholangiocytes (*left*), MMNK-1 cholangiocytes co-cultured with THP-1 monocytes (*centre*) and MMNK-1 cholangiocytes co-cultured with THP-1 macrophages (*right*) were exposed to 1,2-DCP, DCM, and 1,2,3-TCP for 24 hours and the percentage of γ-H2AX_moderate_ (5-54) and γ-H2AX_dense_ (≥55) foci in the 3 types of cholangiocytes was detected by immunochemistry. Data are mean ± SD (*n* = 3). ^*^P < .05, ^**^P < .01, compared with corresponding control group (0 mM), by 1-way analysis of variance (ANOVA) followed by Dunnett’s multiple comparison test.

First, we examined the effects of 1,2-DCP on the expression of γ-H2AX by immunocytochemistry. 1,2-DCP did not change the proportion of γ-H2AX_moderate_ or γ-H2AX_dense_ mono-cholangiocytes. On the other hand, 1,2-DCP dose-dependently increased **γ-**H2AX_dense_ in cholangiocytes of the cholangiocyte/macrophage co-cultures, with a significant change at 0.4 mM (Figure 2, Table 1).

**Table 1:** Effects of various concentrations of 1,2-dichloropropane (1,2-DCP), dichloromethane (DCM), and 1,2,3-trichloropropane (1,2,3-TCP) on number of γ-H2AX foci per cell in mono-cholangiocytes, cholangiocytes co-cultured with undifferentiated monocytes, and cholangiocytes co-cultured with macrophages.

Chemicals and foci type/model	Group	Number of γ-H2AX foci per cell			Simple regression (dose effect) (*P* value)	Multiple regression (*P* value)		
		Concentration of chemicals, mM				Interaction of dose and co-culture	Effect of co-culture	Dose effect
		0	0.1	0.4				
**1,2-DCPmoderate (5-54) foci**						Dose (B to A): −2.4 (0.9) Dose (C to A): −10.7 (0.5)	B to A: 16.9 (<.0001) C to A: 21.7 (<.0001)	19.2 (0.01)
**Monoculture**	A	37.0 ± 6.0	44.0 ± 5.0 (0.34)	48.0 ± 3.0 (0.12)	24 (0.08)			
**Co-culture with THP-1 monocytes**	B	52.3 ± 7.0	63.7 ± 3.2 (0.19)	63.7 ± 6.0 (0.19)	11 (0.29)			
**Co-culture with THP-1 macrophages**	C	59.7 ± 2.6	68.0 ± 5.0 (0.09)	66.0 ± 2.1 (0.17)	15 (0.002)			
**1,2-DCPdense (≥55) foci**						Dose^*^ (B to A): 3.3 (0.37) Dose^*^ (C to A): 11.7 (.004)	—	—
**Monoculture**	A	1.0 ± 0.5	2.0 ± 0.8 (0.48)	3.0 ± 0.5 (0.12)	3 (0.06)			
**Co-culture with THP-1 monocytes**	B	2.3 ± 1.5	3.3 ± 1.5 (0.60)	5.0 ± 1.0 (0.09)	6 (0.04)			
**Co-culture with THP-1 macrophages**	C	4.0 ± 0.5	7.0 ± 0.9 (0.13)	10.0 ± 2.2 (0.007)	14 (0.003)			
**DCMmoderate (5-54) foci**						Dose^*^ (B to A): −24.5 (0.2) Dose^*^ (C to A): −23.7 (0.21)	B to A: 11.4 (.001) C to A: 17.4 (<.0001)	18.4 (0.02)
**Monoculture**	A	37.0 ± 6.0	49.0 ± 3.9 (0.05)	53.0 ± 0.5 (0.01)	34 (0.03)			
**Co-culture with THP-1 monocytes**	B	52.3 ± 7.0	62.7 ± 9.0 (0.29)	59.0 ± 2.4 (0.55)	10 (0.58)			
**Co-culture with THP-1 macrophages**	C	59.7 ± 3.0	67.0 ± 0.5 (0.008)	66.0 ± 0.5 (0.02)	10.8 (0.14)			
**DCMdense (≥55) foci**						Dose^*^ (B to A): 6.5 (0.07) Dose^*^ (C to A): 19.2 (<.0001)	—	—
**Monoculture**	A	1.0 ± 0.5	1.0 ± 0.5 (0.86)	3.0 ± 0.8 (0.10)	4.7 (0.03)			
**Co-culture with THP-1 monocytes**	B	2.3 ± 1.0	4.0 ± 0.8 (0.34)	7.0 ± 1.4 (0.01)	11.3 (0.004)			
**Co-culture with THP-1 macrophages**	C	4.0 ± 0.5	9.0 ± 0.57 (<0.0001)	14.0 ± 0.0 (<0.0001)	23.9 (<.0001)			
**1,2,3-TCPmoderate (5-54) foci**						Dose^*^ (B to A): −78.2 (<.0001) Dose^*^ (C to A): −66.2 (.0002)	—	—
**Monoculture**	A	37.0 ± 6.0	49.0 ± 0.5 (0.04)	66.0 ± 0.9 (0.0005)	70 (.004)			
**Co-culture with THP-1 monocytes**	B	52.3 ± 7.0	56.0 ± 4.0 (0.72)	50.3 ± 4.0 (0.90)	−8.2 (0.53)			
**Co-culture with THP-1 macrophages**	C	59.7 ± 3.0	64.0 ± 2.9 (0.36)	62.0 ± 3.9 (0.64)	3.8 (0.64)			
**1,2,3-TCPdense (≥55) foci**						Dose (B to A): 2.6 (.72) Dose (C to A): 19.6 (.01)	—	—
**Monoculture**	A	1.0 ± 0.5	4.0 ± 0.5 (0.005)	9.0 ± 0.8 (<0.0001)	18.3 (<.0001)			
**Co-culture with THP-1 monocytes**	B	2.3 ± 1.0	6.7 ± 1.2 (0.04)	11.3 ± 1.8 (0.002)	20.89 (0.001)			
**Co-culture with THP-1 macrophages**	C	4.0 ± 0.5	14.0 ± 1.6 (<0.0001)	21.0 ± 0.8 (<0.0001)	37.9 (0.001)			

Data are mean ± SD values (*n* = 3) and *P* value. We performed one-way analysis of variance followed by Dunnett’s multiple comparison test. Interaction between exposure level and co-culture with THP-1 macrophages and monocytes was tested by multiple regression model with moderate (5-54) and dense (≥55) foci numbers of γ-H2AX-positive cells as the dependent variable.

DCM increased **γ-**H2AX_moderate_ in mono-cholangiocytes, with a significant change at 0.4 mM. However, DCM dose-dependently increased **γ-**H2AX_moderate_ and **γ-**H2AX_dense_ in cholangiocytes co-cultured with macrophages, with significant changes at 0.1 and 0.4 mM (Figure 2, Table 1).

1,2,3-TCP increased **γ-**H2AX_moderate_ and **γ-**H2AX_dense_ mono-cholangiocytes, with significant changes at 0.1 and 0.4 mM. 1,2,3-TCP dose-dependently increased **γ-**H2AX_dense_ in cholangiocytes co-cultured with macrophages, with a significant change at 0.4 mM (Figure 2, Table 1).

### 3.3 DCM and TCP dose-dependently increase number of **γ**-H2AX_dense_ in cholangiocytes co-cultured with undifferentiated THP-1 monocytes

We explored the effects of 1,2-DCP, DCM, and 1,2,3-TCP on the expression of γ-H2AX in cholangiocytes cultured with undifferentiated THP-1 monocytes by immunocytochemistry. 1,2-DCP did not change the proportion of γ-H2AX_moderate_ or γ-H2AX_dense_ cholangiocytes of co-cultured cholangiocytes/undifferentiated monocytes (Figure 2, Table 1). On the other hand, DCM increased γ-H2AX_moderate_ but not **γ-**H2AX_dense_ cholangiocytes of co-cultured cholangiocytes/undifferentiated monocytes with a significant change at 0.4 mM. 1,2,3-TCP dose-dependently increased **γ-**H2AX_dense_ in cholangiocytes co-cultured with THP-1 monocytes, with a significant change at 0.4 mM.

### 3.4 Co-culture with macrophages enhances the increase in **γ**-H2AX-positive foci in cholangiocytes induced by all 3 chemical compounds

Multiple regression analysis showed significant interaction of dose and culture type (co-culture with macrophages to monocultures) in 1,2,3-TCP-induced **γ-**H2AX_moderate_ cholangiocytes and 1,2-DCP-, DCM-, and 1,2,3-TCP-induced **γ-**H2AX_dense_ cholangiocytes, confirming the significant effect of co-culture with macrophages on the intensity of the increase in **γ-**H2AX foci induced by the 3 chemical compounds (Table 1). The interaction of dose and culture type (co-culture with monocytes or macrophages to monocultures) was not significant for 1,2-DCP- and DCM-induced **γ-**H2AX_moderate_ cholangiocytes, whereas the effects of culture type (co-culture with monocytes or macrophages to monocultures) and dose were both significant, indicating enhancement of the basal level of **γ-**H2AX_moderate_ cholangiocytes by co-culture with monocytes and macrophages. The interaction of dose and culture type (co-culture with monocytes or macrophages to monocultures) was significant for 1,2,3-TCP-induced **γ-**H2AX_moderate_ cholangiocytes, indicating significant effect of co-culture with monocytes or macrophages on the intensity of 1,2,3-TCP-induced increase in **γ-**H2AX_moderate_ cholangiocytes.

### 3.5 DCP, but not DCM or TCP, decreases pan-nuclear **γ**-H2AX-positive cholangiocytes co-cultured with macrophages

**Figure 3:**
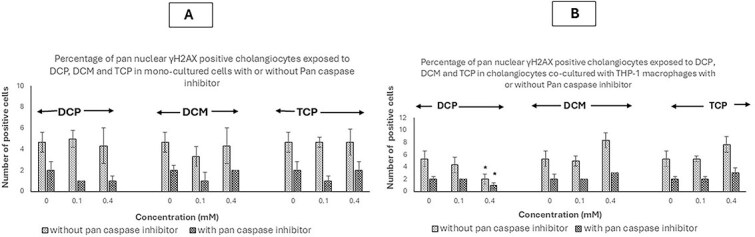
Effects of various concentrations of 1,2-dichloropropane (1,2-DCP), dichloromethane (DCM), and 1,2,3-trichloropropane (1,2,3-TCP) with or without co-treatment with pan-caspase inhibitor on the percentage of pan-nuclear γ-H2AX-positive cholangiocytes in mono-cholangiocytes and cholangiocytes co-cultured with macrophages. (A) Monocultures of MMNK-1 cholangiocytes (*left*) and (B) MMNK-1 cholangiocytes co-cultured with THP-1 macrophages (*right*) were exposed to 1,2-DCP, DCM, and 1,2,3-TCP at concentrations of 0, 0.1, and 0.4 mM, with or without pan-caspase inhibitor, for 24 hours. Data are mean ± SD (*n* = 3). ^*^P < .05, compared with corresponding control (0 mM) group, by 1-way analysis of variance (ANOVA) followed by Dunnett’s multiple comparison test.

Using immunocytochemistry, we compared the effects of 1,2-DCP on pan-nuclear γ-H2AX expression in cholangiocytes versus that in cholangiocytes co-treated with pan-caspase inhibitor. 1,2-DCP dose-dependently decreased the percentage of pan-nuclear γ-H2AX-positive cholangiocytes co-cultured with macrophages, with a significant change at 0.4 mM, but had no effect on mono-cholangiocytes (Figure 3). On the other hand, DCM and 1,2,3-TCP had no effects on pan-nuclear γ-H2AX in mono-cholangiocytes as well as cholangiocytes co-cultured with macrophages (Figure 3). Multiple regression analysis showed no significant interaction of dose and pan-caspase inhibitor, thus validating the additive model of dose and pan-caspase inhibitor (Table 2). The effect of pan-caspase was negative for all 3 compounds, and the effect of dose was negative only for 1,2-DCP, indicating that pan-caspase inhibitor significantly decreased the basal level of pan-nuclear γ-H2AX-positive cholangiocytes co-cultured with macrophages and that 1,2-DCP dose-dependently decreased nuclear γ-H2AX-positive cholangiocytes co-cultured with macrophages.

**Table 2:** Effects of various concentrations of 1,2-dichloropropane (1,2-DCP), dichloromethane (DCM), and 1,2,3-trichloropropane (1,2,3-TCP) on the percentage of pan-nuclear γ-H2AX-positive cholangiocytes in mono-cholangiocytes and cholangiocytes co-cultured with macrophages

Chemicals and model type	Pan nuclear cells type	Concentration (mM)			Simple regression for dose effect (P value)	Multiple regression (P value)		
		0	0.1	0.4		dose-caspase inhibition interaction	pan caspase inhibitor effect	dose effect
1,2-DCP (Monoculture)	Pan nuclear cells without pan caspase inhibitor	4.7 ± 0.9	5.0 ± 0.8 (0.95)	4.3 ± 1.7 (0.95)	−1.2 (0.69)	0.1 (0.97)	−3.2 (<.0001)	−1.2 (0.61)
	Pan nuclear cells with pan caspase inhibitor	2.0 ± 0.8	1.0 ± 0.0 (0.19)	1.0 ± 0.5 (0.42)	−1.03 (0.51)			
1,2-DCP *(Co-culture with THP-1 macrophages)*	Pan nuclear cells without pan caspase inhibitor	5.3 ± 1.2	4.3 ± 1.3 (0.60)	2.0 ± 0.8 (0.04)	−8.2 (0.01)	5.5 (0.08)	−2.1 (0.001)	−8.7 (0.0003)
	Pan nuclear cells with caspase inhibitor	2.0 ± 0.5	2.0 ± 0.0 (0.77)	1.0 ± 0.5 (0.04)	−3.3 (0.03)			
DCM (Monoculture)	Pan nuclear cells without pan caspase inhibitor	4.7 ± 0.9	3.3 ± 0.9 (0.50)	4.3 ± 1.2 (0.95)	0.1 (0.96)	3.8 (0.91)	−2.3 (0.002)	1.2 (0.96)
	Pan nuclear cells with pan caspase inhibitor	2.0 ± 0.5	1.0 ± 0.8 (0.58)	2.0 ± 0.0 (>0.99)	0.5 (0.77)			
DCM *(Co-culture with THP-1 macrophages)*	Pan nuclear cells without pan caspase inhibitor	5.3 ± 1.2	5.0 ± 0.8 (0.94)	8.3 ± 1.2 (0.06)	8.3 (0.01)	−7.2 (0.3)	−3.9 (<.0001)	8.3 (0.001)
	Pan nuclear cells with pan caspase inhibitor	2.0 ± 0.5	2.0 ± 0.0 (0.62)	3.0 ± 0.0 (0.62)	1.2 (0.27)			
1,2,3-TCP (Monoculture)	Pan nuclear cells without pan caspase inhibitor	4.7 ± 0.9	4.7 ± 0.5 (>0.99)	4.7 ± 1.2 (>0.99)	3.4e-15 (1.0)	0.8 (0.77)	−3.0 (<.0001)	2.9 × 10^−15^ (1.0)
	Pan nuclear cells with pan caspase inhibitor	2.0 ± 0.8	1.0 ± 0.5 (0.13)	2.0 ± 0.8 (>0.99)	0.8 (0.61)			
1,2,3-TCP (*Co-culture with THP-1 macrophages)*	Pan nuclear cells without pan caspase inhibitor	5.3 ± 1.2	5.3 ± 0.5 (>0.99)	7.7 ± 1.2 (0.77)	6.3 (0.04)	−4.5 (0.13)	−3.6 (<.0001)	6.3 (0.006)
	Pan nuclear cells with pan caspase inhibitor	2 ± 0.5	2 ± 0.5 (>0.99)	3 ± 0.8 (0.48)	1.8 (0.23)			

Data are mean ± SD values (n = 3) and P value. We performed one-way analysis of variance (ANOVA) followed by Dunnett’s multiple comparison test. The interaction between exposure level and factor of co-culture with THP-1 macrophages was tested using multiple regression model with pan-nuclear cells with and without pan-caspase inhibitor as the dependent variable.

### 3.6 DCP reduces co-expression of TUNEL-positive sites and pan-nuclear **γ**-H2AX in cholangiocytes

**Figure 4:**
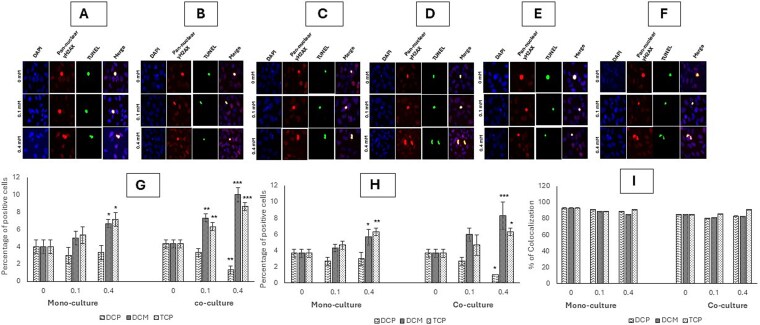
Effects of various concentrations of 1,2-dichloropropane (1,2-DCP), dichloromethane (DCM), and 1,2,3-trichloropropane (1,2,3-TCP) on the percentage of TUNEL (terminal deoxynucleotidyl transferase-mediated dUTP nick end-labeling)-positive and γ-H2AX/TUNEL double-positive mono-cholangiocytes and cholangiocytes co-cultured with macrophages. Images of double staining for γ-H2AX and TUNEL of (A) monocultured cholangiocytes, (B) cholangiocytes co-cultured with THP-1 macrophages treated with 1,2-DCP, (C) monocultured cholangiocytes, (D) cholangiocytes co-cultured with THP-1 macrophages treated with DCM, (E) monocultured cholangiocytes, and (F) cholangiocytes co-cultured with THP-1 macrophages treated with 1,2,3-TCP, at concentrations of 0, 0.1, and 0.4 mM. *Red color*: pan nuclear γ-H2AX-immunostained cholangiocytes; *green color*: TUNEL-stained cholangiocytes; *blue color*: cholangiocyte nuclei stained with 4′,6-diamidino-2-phenylindole (DAPI); merge: colocalization between pan-nuclear γ-H2AX cholangiocytes and TUNEL positive cholangiocytes. Quantitative analysis of the percentages of (G) TUNEL-positive and (H) TUNEL/pan-nuclear γ-H2AX double-positive cholangiocytes in mono-cholangiocytes and cholangiocytes co-cultured with macrophages treated with 1,2-DCP, DCM, and 1,2,3-TCP at concentrations of 0, 0.1, and 0.4 mM. (I) percentage of γ-H2AX/TUNEL double-positive cholangiocytes relative to total TUNEL-positive cholangiocytes in mono-cholangiocytes and cholangiocytes co-cultured with macrophages. Data are mean ± SD (*n* = 3). ^*^P < .05, ^**^P < .01, ^***^P < .001, compared with the corresponding control group (0 mM), by 1-way analysis of variance (ANOVA) followed by Dunnett’s multiple comparison test.

Finally, we used fluorescence microscopy to examine the effects of 1,2-DCP on the localization of pan nuclear γ-H2AX-positive cholangiocytes and TUNEL-positive sites in cholangiocytes. Induction of γ-H2AX and DNA fragmentation detected by TUNEL assay are widely recognized as the hallmarks of apoptosis.^21^^,^^22^ Interestingly, 1,2-DCP dose-dependently decreased the percentage of TUNEL-positive cholangiocytes and cholangiocytes double-positive for pan-nuclear γ-H2AX and TUNEL staining in cholangiocytes co-cultured with macrophages, with a significant change at 0.4 mM, but had no effects in mono-cholangiocytes (Figure 4). In contrast, DCM and TCP dose-dependently increased the percentage of TUNEL-positive mono-cholangiocytes with a significant change at 0.4 mM and increased TUNEL-positive cholangiocytes of cholangiocyte/macrophage co-cultures, with significant changes at 0.1 and 0.4 mM. DCM and TCP increased cholangiocytes double-positive for pan-nuclear γ-H2AX and TUNEL staining, both in mono-cholangiocytes and cholangiocytes co-cultured with macrophages, with a significant change at 0.4 mM (Figure 4). In the presence of 1,2-DCP, DCM, and TCP, the percentage of cholangiocytes positive for pan-nuclear γ-H2AX and TUNEL staining was more than 80% (Figure 4).

## 4. Discussion

Our results showed that only 1,2,3-TCP, at its highest concentration, caused a marked reduction in mono-cholangiocyte viability, indicating that it was the most cytotoxic of the 3 chlorinated hydrocarbons tested in this study. The finding of 1,2-DCP-induced increase in the viability of mono-cholangiocytes is outstanding and in agreement with that reported in our previous study.^16^ Considered together, these findings confirm that 1,2-DCP enhances the proliferation of cholangiocytes.

Proliferation of mono-cholangiocytes observed in the present study may explain the biliary hyperplasia reported previously in non-cancerous liver tissue of 1,2-DCP-exposed workers afflicted with bile duct cancer.^5^ It may also explain our recent findings of increased bile duct cell proliferation in mice exposed for 5 weeks to 1,2-DCP inhalation.^23^

Macrophages release various factors, such as tumor necrosis factor α (TNF-α) and nitric oxide (NO) during inflammation, which can induce DNA damage, promote mutagenesis, and drive genomic instability, ultimately contributing to tumorigenesis.^24^^,^^25^ By eliciting persistent macrophage inflammation and increasing TNF-α levels, 1,2-DCP activates the nuclear factor κB (NF-κB) pathway, resulting in aberrant activation-induced cytidine deaminase (AICD) expression.^17^ The combined outcome of increased proliferation and macrophage-derived inflammatory signals, such as TNF-α, creates a microenvironment that facilitates tumorigenesis. Dichloromethane (DCM) itself has been shown experimentally to increase mutagenic DNA damage, oxidative adducts (8-OHdG, 8-nitroguanine), and transformation potential in MMNK-1 cholangiocytes and to alter the expression of genes involved in oxidative stress and inflammation—providing direct evidence that DCM can act on cholangiocytes to promote genotoxic and pro-carcinogenic changes.^25^

Examination of 1,2-DCP-, DCM-, and 1,2,3-TCP-induced γ-H2AX foci in mono-cholangiocytes and cholangiocyte/macrophage co-cultures showed that 1,2,3-TCP induced the most severe DNA damage. In addition, DNA damage induced by the 3 chemical compounds was enhanced in cholangiocytes co-cultured with macrophages. The observed increase in γ-H2AX foci in 1,2-DCP-exposed cholangiocytes co-cultured with macrophages is also in agreement with the results of our previous studies.^26^ Co-culture with undifferentiated monocytes also enhanced 1,2,3-TCP-induced DNA damage, though to a lesser extent than co-culture with macrophages, and only increased the basal level of DCP- or DCM-induced **γ-**H2AX_moderate_ cholangiocytes. Considered together, these results suggest that differentiation of monocytes increases the ability of monocytes to enhance DNA damage induced by all 3 chemical compounds in cholangiocytes.

It is also reported that 1,2-DCP generates γ-H2AX, a marker of DNA damage, in cultured human hepatocytes and cholangiocytes and in the liver of mice.^27^ Our study showed similar increases in γ-H2AX foci in cholangiocytes treated with DCM and 1,2,3-TCP. The finding of 1,2-DCP-induced DNA damage is consistent with increased expression of γ-H2AX in non-neoplastic biliary epithelial sites of workers exposed to 1,2-DCP who developed bile duct cancer.^6^ γ-H2AX is a well-established marker of DNA double-strand breaks (DSBs), which constitute a principal source of genomic instability—a hallmark of cancer. Persistent γ-H2AX expression indicates unrepaired or misrepaired DNA lesions, which can indirectly foster tumor initiation and progression by increasing mutation rates, chromosomal aberrations, and genomic instability.^28^

The study showed that only DCP decreased pan-nuclear γ-H2AX-positive cholangiocytes, TUNEL-positive cholangiocytes, and γ-H2AX and TUNEL double-positive cholangiocytes in cholangiocyte/macrophage co-cultures. The ~50% reduction in pan-nuclear γ-H2AX-positive cholangiocytes by the pan-caspase inhibitor indicates that apoptotic signaling contributes, at least in part, to the pan-nuclear γ-H2AX immunostaining. Pan-nuclear γ-H2AX-positive cholangiocytes—cells in which the entire nucleus is uniformly stained with γ-H2AX antibody—are considered indicative of early apoptosis.^29^^,^^30^ Apoptosis is a cellular process that eliminates cells with damaged DNA,^31^ and reduced apoptosis is associated with tumorigenesis.^32^ A reduction in pan-nuclear γ-H2AX-positive cells suggests diminished apoptosis, which may allow damaged cells to survive and thereby contribute to cancer progression.^22^

1,2-DCP, but neither DCM nor 1,2,3-TCP, dose-dependently decreased pan-nuclear γ-H2AX-positive cholangiocytes treated with and without caspase inhibitor. Co-treatment with the pan-caspase inhibitor reduced the number of spontaneously generated pan-nuclear γ-H2AX-positive cholangiocytes, suggesting the involvement of caspase in the generation of pan-nuclear γ-H2AX-positive cholangiocytes. Furthermore, the double staining for TUNEL and pan-nuclear γ-H2AX in 1,2-DCP cholangiocytes, identified in more than 80% of such cholangiocytes, strongly suggests a link between pan-nuclear γ-H2AX positivity and apoptosis. These results add support to the conclusion made in previous studies that pan-nuclear γ-H2AX-positive cholangiocytes reflect the early stage of apoptosis.^29^^,^^30^

Finally, our study showed that 1,2-DCP dose-dependently decreased the percentage of TUNEL-positive cholangiocytes/macrophages whereas DCM and TCP dose-dependently increased the percentage of TUNEL-positive cholangiocytes in both mono-cholangiocytes and cholangiocytes/macrophages. These results suggest that 1,2-DCP exhibits anti-apoptotic properties in cholangiocytes co-cultured with macrophages.

## 5. Conclusions

Our study demonstrated that among the 3 tested chlorinated hydrocarbons (1,2-DCP, DCM, and 1,2,3-TCP), the latter was the strongest inducer of DNA damage in mono-cholangiocytes and cholangiocyte/macrophage co-cultures. While our study confirmed the previously reported finding that macrophages enhance DCP-induced DNA damage in cholangiocytes, it also highlighted the role of macrophages in enhancing DCM- and 1,2,3-TCP-induced DNA damage in cholangiocytes. Among the 3 chemical compounds tested in this study, only 1,2-DCP induced a concentration-dependent proliferative effect in monocultured cholangiocytes and exhibited anti-apoptotic properties in cholangiocyte/macrophage co-cultures. These findings suggest that 1,2-DCP displays key carcinogenic features—namely, the promotion of cell proliferation and inhibition of apoptosis—setting it apart from DCM and 1,2,3-TCP.

## Supplementary Material

Supplementary_materials_uiag029

## Data Availability

Data underlying this article will be shared on reasonable requests to the corresponding author.
