# Supplementary material for: 1,2-Dichloropropane, but not dichloromethane or trichloropropane, reduces apoptosis of human cholangiocytes co-cultured with macrophages
Source: J Occup Health. 2026 Jun 1;68(1):uiag029. doi: 10.1093/joccuh/uiag029 (PMC13335648; doi:10.1093/joccuh/uiag029)
Supplement: Supplementary_materials_uiag029 [file supplementary_materials_uiag029.zip › Supplementary Figure caption .docx]

**Supplementary Figure 1S. Distribution of cholangiocyte number by fγ-H2AX-positive foci in cholangiocyte-macrophage co-cultures exposed to vehicle for 24 hours.**

MMNK-1 cholangiocytes co-cultured with THP-1 macrophages were exposed to vehicle 0.1% DMSO for 24 hours.
