# Supplementary figures and images for: 1,2-Dichloropropane, but not dichloromethane or trichloropropane, reduces apoptosis of human cholangiocytes co-cultured with macrophages
Source: J Occup Health. 2026 Jun 1;68(1):uiag029. doi: 10.1093/joccuh/uiag029 (PMC13335648; doi:10.1093/joccuh/uiag029)

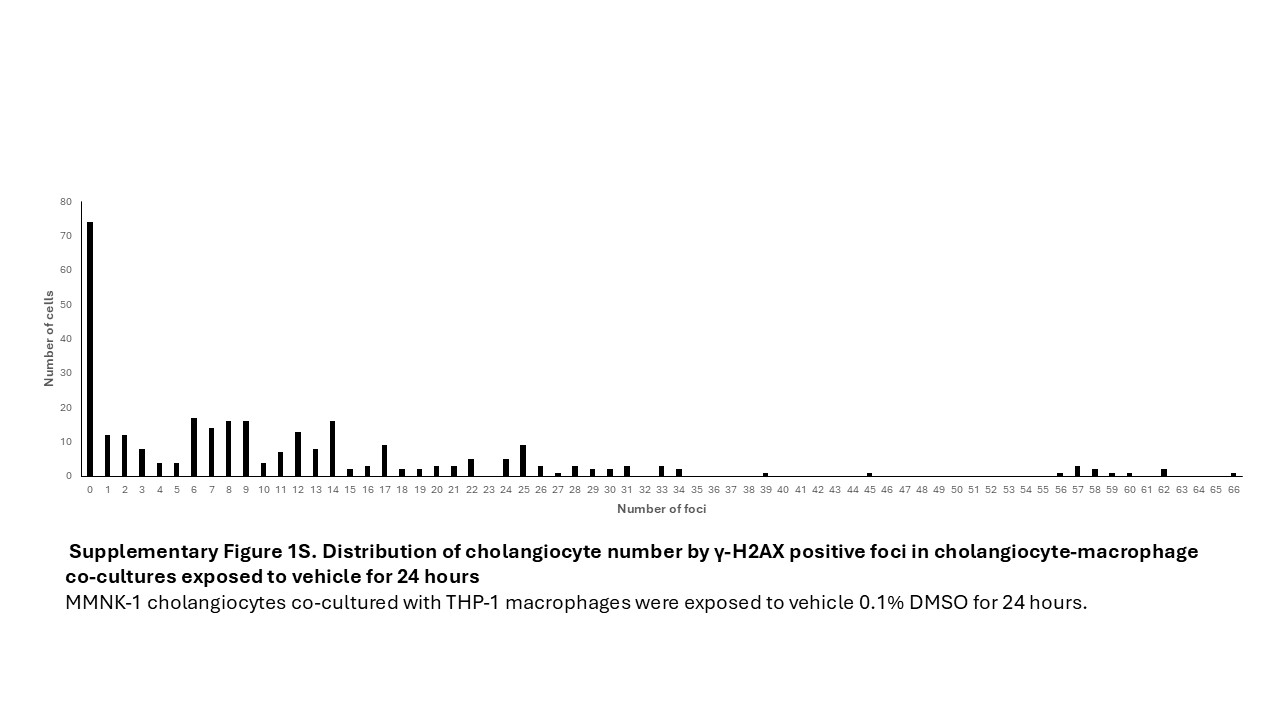

Supplement: Supplementary_materials_uiag029 [file supplementary_materials_uiag029.zip › Rahman M, et al., supplementary figure 1S.jpg]
